# *Bacillus paralicheniformis* 809 and *Bacillus subtilis* 810 support in vitro intestinal integrity under hydrogen peroxide and deoxynivalenol challenges

**DOI:** 10.1093/tas/txae061

**Published:** 2024-04-13

**Authors:** Erik J Boll, Giuseppe Copani, Bruno I Cappellozza

**Affiliations:** Novonesis, Hørsholm 2970, Denmark; Novonesis, Hørsholm 2970, Denmark; Novonesis, Hørsholm 2970, Denmark

**Keywords:** *B. paralicheniformis*, *B. subtilis*, deoxynivalenol, hydrogen peroxide, intestinal integrity

## Abstract

We designed and conducted two in vitro experiments to evaluate the effects of two *Bacillus* spp. probiotics on gut barrier integrity using the transepithelial electrical resistance (**TEER**) assay under two different challenge models. In Exp. 1, intestinal epithelial cells received or not (**CON**) *B. paralicheniformis* 809 (**BLI**) or *B. subtilis* 810 (**BSU**) at a rate of 1 × 10^8^ colony forming units (**CFU**)/transwell. Two hours after treatment application (CON, BLI, or BSU), 5 mM of the reactive oxygen species hydrogen peroxide, mimicking mucosal oxidative stress, was added alone (**HYP**) or with each of the *Bacillus* spp. (HYP + BLI or HYP + BSU). In Exp. 2, cells were assigned to the same treatments as in Exp. 1 (CON, BLI, and BSU), or mycotoxin deoxynivalenol (**DON**), which was added alone or in combination with BLI or BSU, resulting in another two treatments (DON + BLI and DON + BSU). Transepithelial electrical resistance was measured for 14 h postchallenge. In Exp. 1, a treatment × hour interaction was observed for TEER (*P* < 0.0001). Adding BLI and BSU resulted in greater TEER values vs. CON for most of the experimental period (*P* < 0.02), whereas HYP reduced mean TEER and area under the curve (**AUC**), while increasing the amount of sugar that translocated through the monolayer cells (*P* < 0.001). A treatment × hour interaction was also observed in Exp. 2 (*P* < 0.0001), as DON led to an immediate and acute drop in TEER that lasted until the end of the experimental period (*P* < 0.0001). Both BLI and BSU alleviated the DON-induced damaging effects on the integrity of intestinal epithelial cells, whereas both *Bacillus* spp. alleviated the damage caused by DON alone and the proportion of sugar that translocated through the monolayer cells was not different between CON and DON + BLI (*P* = 0.14) and DON + BLI and DON + BSU (*P* = 0.62). In summary, both *Bacillus* spp. strains (*B. paralicheniformis* 809 and *B. subtilis* 810) were able to counteract the damaging effects of the challenge agents, hydrogen peroxide and deoxynivalenol, on gut barrier integrity.

## Teaser

Both challenges, hydrogen peroxide and deoxynivalenol, lead to a loss in the integrity of the intestinal epithelial cells.

Adding the *Bacillus* spp. alleviated the damage caused by the challenges, supporting an integral state of the intestinal epithelial cells under an in vitro setting.

## INTRODUCTION

Bacilli are spore-forming bacteria that can tolerate the most challenging environments (Bernardeau et al., 2017) and exposure to, e.g., wide pH variations, high temperatures, and variable degrees of moisture during feed preparation (Cappellozza et al., 2023a). Among the modes of action of *Bacillus*-based direct-fed microbials (**DFM**), support of the gastrointestinal tract (**GIT**) health of the host is highlighted (Luise et al., 2022). More specifically, *Bacillus* spp. stimulate the in vitro production and release of mucins from goblet-like intestinal epithelial cells, inhibit the growth of potentially harmful bacteria (i.e., enterotoxigenic *Escherichia coli* F4 and *Clostridium perfringens* type A), stimulate biofilm formation, and inhibit the adhesion of enterotoxigenic *E. coli* to intestinal epithelial cell monolayers (Santano et al., 2020; Segura et al., 2020). Hence, the interest in using Bacilli as DFM is increasing with the goal to support the GIT health of ruminants.

The GIT of ruminants is challenged with different stressors, including potentially harmful bacteria, mycotoxins, reactive oxygen species (**ROS**), endotoxins, and pro-inflammatory cytokines that may lead to the occurrence of the hyperpermeable intestine syndrome (Copani et al., 2021; Boll et al., 2024; de Gregorio et al., 2023). This syndrome, also known as leaky gut, alters the GIT microbiota (O’Hara, et al., 2020), triggering an inflammatory response that leads to production losses in beef and dairy cattle (Cooke, 2017; Kvidera et al., 2017a, b; Briggs et al., 2021). Mycotoxins, such as deoxynivalenol (**DON**), and ROS accumulation may also lead to dysbiosis and increased permeability of the GIT, leading to damages of the gut epithelium (Antonissen et al., 2014; Mazzoli et al., 2019). Therefore, it is of interest to the ruminant production segment to evaluate feeding strategies that support the integrity of intestinal epithelial cells when challenged with stressors, such as DON and ROS. Based on this rationale, we hypothesized that adding *Bacillus* spp. would support gut barrier integrity under conditions mimicking oxidative stress or ingestion of mycotoxins, such as DON. Hence, our objective was to evaluate the effects of adding *Bacillus* spp. on the in vitro integrity of epithelial intestinal cells challenged with hydrogen peroxide (Exp. 1) or DON (Exp. 2).

## EXPERIMENTAL PROCEDURE

### Cell Culture

The human cancer-derived epithelial intestinal cell lines Caco-2 (ACC 169, DSMZ, passages 5 to 20) were maintained in Dulbecco’s Modified Eagle Medium (**DMEM**, Gibco, Thermo Fischer Scientific, Roskilde, Denmark) supplemented with non-essential amino acids (Biowest, Nuaillé, France), penicillin streptomycin-Amphotericin B Solution (Biological Industries, Kibutz Beit-Haemek, Israel), and 10% fetal bovine serum (Gibco, Roskilde) at 37 °C and 5% of carbon dioxide (**CO**_**2**_). For the transepithelial electrical resistance (**TEER**) assay, Caco-2 cells were seeded on 1.12-cm^2^ transwells (0.4 μm pore size; Corning Labware, Warsaw, Poland) at 5 × 10^4^ cells/insert. The culture medium was changed every 3 to 4 d and the cells were utilized after 20 to 22 d of culture, by which time they had reached a confluent, polarized, and differentiated state. Caco-2 cells are derived from colon carcinoma and are widely used as a model for intestinal epithelial cells (Sambuy et al., 2005; Ferruzza et al., 2013). Due to their close resemblance to enterocytes, they have been increasingly used as an in vitro model for high-throughput screening assays in the last decades for testing intestinal absorption of different compounds (Sambuy et al., 2005; Van Breemen and Li, 2005).

### Bacterial Strains and Growth Conditions

In both experiments reported below, *Bacillus licheniformis* 809 (**BLI**) and *B. subtilis* 810 (**BSU**) were used as the DFM bacterial strains. For growth in liquid culture, all *Bacillus* spp. strains were cultured in a Luria Bertani broth (**LB**; Sigma-Aldrich, London, UK). All strains were incubated at 37 °C, with shaking at 200 rpm. Then, *Bacillus* strains were subsequently diluted 1:100 in a LB broth and grown with shaking for 3 h to an early exponential phase. For growth on a solid medium, *Bacillus* strains were grown on Trypticase Soya Agar (**TSA**; Sigma-Aldrich) plates and incubated at 37 °C.

### Transepithelial Electrical Resistance Assay

Caco-2 cell monolayers were equilibrated overnight in an antibiotic-free cell culture medium in a CellZscope2 system (NanoAnalytics GmbH, Münster, Germany). On the day of the experiment, bacterial strains grown as described above were washed twice using Hanks balanced salt solution (HBSS, Gibco, Roskilde) and resuspended in antibiotic-free cell culture media. The OD_600nm_-normalized bacteria were then added to the apical compartments of the cell monolayers at a concentration of approximately 1 × 10^8^ colony forming units (**CFU**)/transwell for both *Bacillus* spp., resulting in four treatments: (1) no Bacilli or hydrogen peroxide addition to the cells (**CON**), (2) addition of hydrogen peroxide at 5 mM in the apical compartment of each transwell (**HYP**), (3) HYP + *B. licheniformis* 809 (HYP + BLI), or (4) HYP + *B. subtilis* 810 (HYP + BSU). The HYP was added 2 h after adding the Bacilli. For Exp. 2, the mycotoxin deoxynivalenol (DON) was used as the in vitro challenge, resulting in the following treatments: (1) no Bacilli or DON addition to the cells (CON), (2) addition of DON at 75 µM to the apical and basolateral compartment of the cells in each transwell (DON), (3) DON + *B. licheniformis* 809 (DON + BLI), or (4) DON + *B. subtilis* 810 (DON + BSU). Regardless of the experiment, the volume of media added to the basolateral and apical compartments were 1,650 and 750 µL, respectively. Moreover, adding DON to both the apical and basolateral sides of the cells is likely to mimic an in vivo situation, as DON is absorbed in the upper parts of the small intestine and likely to be secreted into the gut lumen (Akbari et al. 2014). Moreover, the dosages of BLI, BSU, HYP, and DON have been chosen based on previous in vitro dose–response assays in our lab (data not shown). In both experiments, hourly TEER measurements were carried out for up to 14 h postchallenge administration. Results are expressed as relative to baseline values before the addition of the HYP (Exp. 1), DON (Exp. 2), and *Bacillus* spp. to the cell monolayers. In both experiments, following the period of TEER evaluation, the area under the curve (**AUC**) was also calculated. It is noteworthy mentioning that in the TEER assay, values above 100 indicate a stronger bond of the cells, whereas values below 100 indicate a leaky gut state (Srinivasan et al., 2015). In both experiments, a minimum of nine independent replicates per treatment were evaluated and within experiments, replicates were analyzed on the same day.

### Fluorescein Isothiocyanate Dextran Translocation Assay

In the beginning of each experiment, fluorescein isothiocyanate (FITC) dextran-20 kDa (FD20; Sigma-Aldrich) was added to the apical compartments to a final amount of 400 μg per transwell. Then, at the end of the experiment, the amount of FD20 translocated to the basolateral compartment was quantified by measuring the fluorescent signal of the basolateral supernatants (490 nm emission/520 nm excitation) using a microplate reader (Synergy H1; BioTek). From this, the percentage of translocated FD20 relative to the apically added baseline level was quantified.

### Statistical Analysis

Data from both experiments were analyzed separately and not across them, because of (1) variations in TEER results between monolayers (baseline resistance, 300 to 350 Ω × cm^2^) and (2) the goal here was not to compare different challenges leading to leaky gut, but the efficacy of both Bacilli strains in supporting the integrity of intestinal cells upon both challenges. All TEER and FITC data were analyzed with PROC MIXED of SAS (version 9.4; SAS Inst., Cary, NC) and results were expressed as the mean ± SEM of an individual experiment. Moreover, data were analyzed over time (for TEER) and, therefore, treatment × hour interactions were also tested and reported in the manuscript. Lastly, the AUC data were calculated and pooled with PRISM (GraphPad Software, Boston, MA), and the values analyzed with SAS (SAS Inst.). Significances were set at *P* ≤ 0.05, whereas tendencies were denoted if 0.05 < *P* ≤ 0.10.

## RESULTS

### Experiment 1

**Figure 1.**
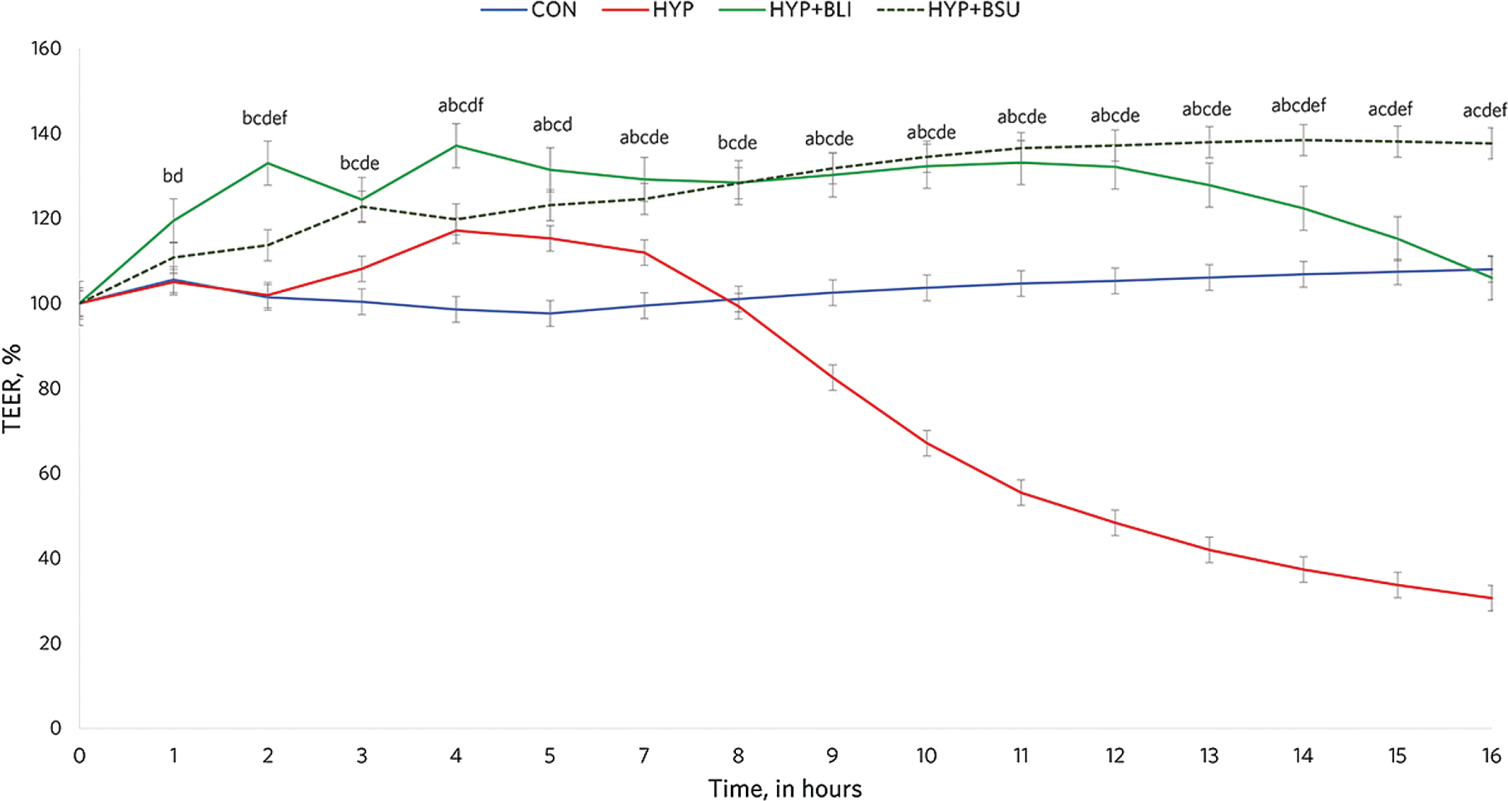
Transepithelial electrical resistance across intestinal epithelial cell monolayers exposed or not (CON) to hydrogen peroxide (5 mM; HYP) alone or in combination with 1 × 10^8^ CFU/mL of *Bacillus licheniformis* 809 (HYP + BLI) or 1 × 10^8^ CFU/mL of *B. subtilis* 810 (HYP + BSU). A treatment × hour interaction was observed (*P* < 0.0001). The following comparisons denote differences at *P* ≤ 0.05: ^a^CON vs. HYP; ^b^CON vs. HYP + BLI; ^c^CON vs. HYP + BSU; ^d^HYP vs. HYP + BLI; ^e^HYP vs. HYP + BSU; ^f^HYP + BLI vs. HYP + BSU.

As expected, a treatment × hour interaction was observed for TEER (*P* < 0.0001; Fig. 1). Following HYP addition to the cells at 2 h, an increasing drop in TEER values was observed in HYP vs. CON from 8.7 h to the end of the evaluation period (*P* < 0.0001; Fig. 1). On the other hand, adding BLI and BSU resulted in increased TEER values vs. CON for most of the experimental period (*P* < 0.02), whereas a drop in TEER was observed for HYP + BLI at 15.2 and 16.3 h that was not different from CON (*P* ≥ 0.20). Therefore, adding HYP reduced mean TEER when compared with the other treatments (*P* < 0.0001) and both BLI and BSU supported the integrity of the cells by increasing TEER vs. CON and HYP alone (*P* < 0.0001; Table 1). Similar treatment effects were observed for both AUC and the % of FD20 that was translocated across the epithelial cell monolayers (*P* < 0.001; Table 1).

**Table 1.** Mean transepithelial electrical resistance, area under the curve, and FD20 translocation from epithelial cells receiving the different treatments in Exp. 1 and 2^1^^,^^2^

Item	TEER, %	AUC	FD20, %
Experiment 1^3^			
CON	103.0^b^	86.2^b^	0.11^b^
HYP	78.5^c^	−302.2^c^	3.35^a^
HYP + BLI	125.1^a^	469.4^a^	0.53^b^
HYP + BSU	127.2^a^	490.7^a^	0.06^b^
SEM	2.62	54.49	0.627
*P*-value	<0.0001	<0.0001	<0.001
Experiment 2^4^			
CON	100.1^a^	1.5^a^	0.08^c^
DON	59.3^d^	−569.0^d^	2.20^a^
DON + BLI	84.6^b^	−204.8^b^	0.45^bc^
DON + BSU	79.2^c^	−289.7^c^	0.88^b^
SEM	1.46	24.44	0.193
*P*-value	<0.0001	<0.0001	<0.0001

^1^Different letters within each experiment denote significance at *P* < 0.05.

^2^The TEER assays were conducted over a period of 16 and 14 h for Exp. 1 and 2, respectively.

^3^Treatments were (1) a control without the addition of the challenge or the direct-fed microbial (CON), (2) 5 mM of hydrogen peroxide/transwell (HYP), (3) HYP plus 1.0 × 10^8^ CFU/transwell of *Bacillus licheniformis* 809 (HYP + BLI), or (4) HYP + 1.0 × 10^8^ CFU/transwell of *B. subtilis* 810 (HYP + BSU).

^4^Treatments were (1) a control without the addition of the challenge or the direct-fed microbial (CON), (2) 75 µM of DON/transwell (DON), (3) DON plus 1.0 × 10^8^ CFU/transwell of *B. licheniformis* 809 (DON + BLI), or (4) DON plus 1.0 × 10^8^ CFU/transwell of *B. subtilis* 810 (DON + BSU).

### Experiment 2

**Figure 2.**
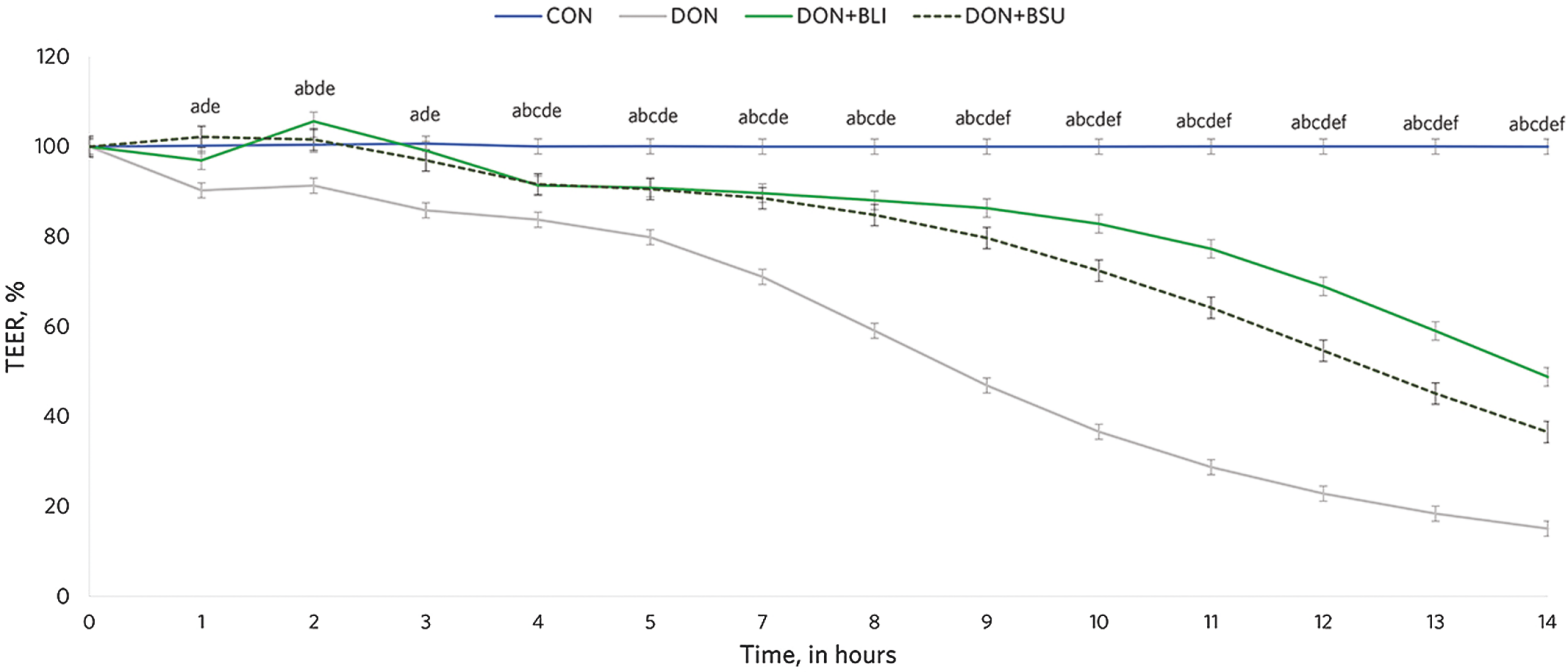
Transepithelial electrical resistance across epithelial cell monolayers exposed or not (CON) to deoxynivalenol (75 µM; DON) alone or in combination with 1 × 10^8^ CFU/mL of *Bacillus licheniformis* 809 (HYP + BLI) or 1 × 10^8^ CFU/mL of *B. subtilis* 810 (DON + BSU). A treatment × hour interaction was observed (*P* < 0.0001). The following comparisons denote differences at *P* ≤ 0.05: ^a^CON vs. DON; ^b^CON vs. DON + BLI; ^c^CON vs. DON + BSU; ^d^DON vs. DON + BLI; ^e^DON vs. DON + BSU; ^f^DON + BLI vs. DON + BSU.

A treatment × hour interaction was also observed in Exp. 2 (*P* < 0.0001; Fig. 2), as DON led to an immediate and acute drop in TEER that lasted until the end of the experimental period (*P* < 0.0001). Both BLI and BSU alleviated the DON-induced damaging effects on the integrity of intestinal epithelial cell monolayers, but TEER values were also lower than CON from 4.4 to 14.1 h (*P* < 0.01). Moreover, BLI yield greater TEER results than BSU from the last part of the experimental period (8.7 to 14.1 h; *P* ≤ 0.04; Fig. 2). As reported above, DON by itself led to an acute and immediate damaging effect that resulted in the lowest mean TEER, AUC, and greatest FD20 translocation vs. CON and addition of both Bacilli to DON (*P* < 0.001; Table 1). On the other hand, including the *Bacillus* spp. (BLI or BSU) with DON alleviated the damage caused by DON alone, yielding TEER and AUC values that were lower than CON. Moreover, BLI resulted in greater TEER and AUC than BSU (*P* = 0.03), whereas FD20 proportion was not different between CON and DON + BLI (*P* = 0.14) and DON + BLI and DON + BSU (*P* = 0.62; Table 1).

## DISCUSSION

The main goal of these experiments was to evaluate whether *B. licheniformis* 809 and *B. subtilis* 810 could support the in vitro integrity of intestinal epithelial cell monolayers upon a challenge with hydrogen peroxide (Exp. 1) or deoxynivalenol (Exp. 2). Others have demonstrated that leaky gut occurrence can trigger an inflammatory response that ultimately leads to significant reductions in dry matter intake and milk yield of lactating dairy cows (Kvidera et al., 2017a), as well as reductions in average daily gain that leads to lighter carcasses at slaughter of beef cattle (Briggs et al., 2021). Therefore, evaluating alternatives that support the integrity of the GIT and its cells, while also reducing or alleviating the occurrence of leaky gut, and maintaining adequate health and performance of the beef and dairy cattle herd are imperative to support the profitability of livestock operations. *Bacillus* spp. have been included in commercially available DFM products in several livestock species (Luise et al., 2022), whereas in the United States, *B. subtilis* is one of the most common bacterial species included in commercial products for poultry (Joerger and Ganguly 2017; Elishavili et al., 2019) as an alternative to antibiotics (Jiang et al., 2021). Among the benefits of *Bacillus* spp. on GIT function and health of the host, mucin production (Santano et al., 2020), microbiome modulation (Linde et al., 2023), intestinal cell development (Sun et al., 2023), competitive inhibition vs. potentially harmful bacteria (Santano et al., 2020), an improvement on mRNA expression of tight junction proteins (Sun et al., 2023), and antioxidant activity (Ramlucken et al., 2020) have been highlighted. In fact, feeding a combination of Bacilli improved the function of intestinal cell monolayers in the jejunum and duodenum of *C. perfringens*-challenged broilers, which was measured by the villus height to crypt depth ratio (Konieczka et al., 2022).

ROS, such as hydrogen peroxide, are highly oxidative substances derived from oxygen and have been recognized as one of the key players impacting the lower GIT environment (Aviello and Knaus, 2017). These compounds are produced as part of the mitochondrial respiratory pathway and following phagocytic activity of innate immune cells (Ikeda et al., 2023). Under homeostasis, production of ROS is considered to be under a normal threshold and is tightly controlled by specific endogenous enzymes (i.e., superoxide dismutase and glutathione peroxidase), but ROS production dramatically changes following episodes of neuroendocrine and environmental stress, as well as disease (Sordillo and Aitken, 2009; Gu et al., 2023). The combined increased production and release of ROS into the circulation increase its concentration to a level that the body cannot mitigate. Therefore, excessive inflammation, immune dysfunction, and cellular macromolecule damage might be observed (Fulda et al., 2010; Sottero et al., 2018), impairing the health and performance of beef and dairy cattle (Gu et al., 2023). In piglets, feeding *B. licheniformis* S6 improved the activity of antioxidant enzymes and reduced serum malondialdehyde concentrations (Sun et al., 2023), likely supporting the function and health of the GIT. Our in vitro results (Exp. 1) demonstrated that (i) hydrogen peroxide damages the integrity of intestinal epithelial cell monolayers, leading to the occurrence of leaky gut and (ii) adding *B. licheniformis* 809 or *B. subtilis* 810 supported the integrity of the cells over time. It is interesting to note the TEER effects observed over time, as hydrogen peroxide reduced TEER as the experiment progressed, whereas Bacilli supported the integrity of the cells. Nonetheless, additional studies are warranted to understand if the combination of both strains will yield similar or better results than the current ones evaluating the single DFM strains. In fact, despite a lack of studies evaluating the intestinal integrity of both strains under an in vivo ruminant setting, feeding a combination of *B. licheniformis* 809 and *B. subtilis* 810 has resulted in greater nutrient digestibility (Pan et al., 2022; Cappellozza et al., 2023b; Oyebade et al., 2023), heavier calves at weaning (Kowalski et al., 2009), greater milk solids yield, milk yield, and efficiency (Kritas et al., 2006; Cappellozza et al., 2024; Oyebade et al., 2023), and greater feed efficiency in feedlot beef cattle (Dias et al., 2022). Moreover, while our in vitro findings suggest beneficial impacts of these two probiotic strains intestinal mucosal oxidative stress, it will be interesting to investigate whether they may also impact systemic oxidative stress as suggested for other strains of Bacilli (Crescenzo et al., 2017; Zhang et al., 2021).

Mycotoxins are produced by fungal species and have often been found in cereal feedstuffs offered to ruminants, such as corn, wheat, barley, rye, and oats (Magan and Aldred, 2007). Several mycotoxins have been identified at this point, with DON identified as one of the most important and observed types in ruminant feed, being produced by two strains of Fusarium during pre- or post–crop harvesting. Deoxynivalenol disrupts the integrity of bovine epithelial cells structure and function, inhibiting its proliferation and differentiation, leading to cellular apoptosis, whereas clinical signs in the animals include anorexia, diarrhea, and performance losses (Akbari et al., 2014; Bailey et al., 2019). Supporting previous research, our results indicate that DON leads to an acute and immediate effect on the integrity of in vitro intestinal epithelial cells, as TEER values began declining already at 1-h post-DON addition to roughly 15% by the end of the experimental period. On the other hand, adding *B. licheniformis* or *B. subtilis* alleviated the DON-stimulated TEER reduction, and to the best of our knowledge, this is one of the first data demonstrating such effect with Bacilli. We speculate that the positive effects on alleviating TEER reduction are likely associated with the support of tight junction proteins (Konieczka et al., 2022), a greater mucin production when mucin-producing cell types are evaluated (Santano et al., 2020), and a thicker mucous layer that could reduce the access of DON to the cells, competitive exclusion resulting in reduction in the damage of DON to the intestinal cells (Copani et al., 2020; Santano et al., 2020), and biofilm formation (Segura et al., 2020). Considering that cereal grains and their forages are the major feedstuffs included in a ruminant diet (Samuelson et al., 2016; Silvestre and Millen, 2021), there is a likelihood that DON will be fed through the diet and, therefore, consumed by the animals daily during their productive stages (beef and dairy). As an example, Gallo et al. (2020) reported a reduction in feed efficiency, milk yield, dry matter, and NDF digestibility of dairy cows offered 340.5 µg of DON and 127.9 µg of fumonisin/kg of dry matter. Hence, it is imperative to provide technologies that support the integrity of the epithelial cells when animals are routinely challenged with DON in the feedlot and/or dairy farm.

The treatment × time interactions observed for both experiments are novel and interesting, demonstrating that continuous exposure to stressors (hydrogen peroxide and DON) resulted in additional damaging effects on the cells under an in vitro setting. Moreover, including Bacillus spp. supported the integrity of the cells when challenged with ROS, but alleviated the damaging effects caused by DON. Whether these effects are replicated using in vivo ruminant models and/or under-production settings is still to be evaluated, but our data warrant further research efforts in this area of interest.

In summary, hydrogen peroxide and deoxynivalenol compromised the integrity of intestinal epithelial cells in an in vitro setting. The inclusion of *Bacillus* spp. (*B. licheniformis* 809 or *B. subtilis* 810) supported the in vitro integrity of intestinal epithelial cell monolayers upon a hydrogen peroxide challenge and alleviated the loss of epithelial cell integrity following the deoxynivalenol challenge. Nonetheless, additional research efforts are warranted to show such benefits under different production settings often encountered in beef and dairy cattle, as well as the benefits of adding the two *Bacillus* spp. evaluated herein on supporting the integrity of intestinal cells in vivo.
